# Assessment of the Presence of Resistance Genes Detected from the Environment and Selected Food Products in Benin

**DOI:** 10.1155/2021/8420590

**Published:** 2021-02-04

**Authors:** Victorien Dougnon, Vincentia Marie Camille Houssou, Eugénie Anago, Chimène Nanoukon, Jibril Mohammed, Jerrold Agbankpe, Hornel Koudokpon, Birikissou Bouraima, Esther Deguenon, Kafayath Fabiyi, Marie Hidjo, Fidélia Djegui, Lamine Baba-Moussa, Martin Pépin Aïna

**Affiliations:** ^1^Research Unit in Applied Microbiology and Pharmacology of Natural Substances, Research Laboratory in Applied Biology, Polytechnic School of Abomey-Calavi, University of Abomey-Calavi, P. O. Box 2009, Cotonou, Benin; ^2^Laboratory of Monitoring and Environmental Studies, Ministry of the Living Environment and Sustainable Development, Benin; ^3^Laboratory of Molecular Biology and Bioinformatics Applied to Genomics, National School of Biosciences and Applied Biotechnologies, National University of Sciences, Technologies, Engineering and Mathematics, Abomey, Benin; ^4^Department of Microbiology, Parasitology and Biotechnology, College of Veterinary Medicine and Biomedical Sciences, Sokoine University of Agriculture, Morogoro, Tanzania; ^5^Laboratory of Veterinary Diagnosis and Serosurveillance of Parakou, Ministry of Agriculture, Livestock and Fisheries, Parakou, Benin; ^6^Laboratory of Biology and Molecular Typing in Microbiology, Faculty of Science and Technology, University of Abomey-Calavi, UAC, P. O. Box 1604, Cotonou, Benin

## Abstract

Gram-negative bacilli can spread from the environment and through food products. This study aimed to characterize ESBL production and virulence genes from multidrug-resistant Gram-negative bacilli isolated from specimen collected from the environment, kitchen, and food products. A total of 130 samples were collected at local markets in seven different communities in Benin (Abomey-Calavi, Ouidah, Bohicon, Abomey, Parakou, Djougou, and Grand-Popo). Samples were cultured on McConkey and ChromID^™^ ESBL agar plates. The isolates were identified by the API 20E gallery. An antibiotic susceptibility test was carried out, and the detection of ESBL production and virulence-associated genes was carried out by Polymerase Chain Reaction (PCR). The data collected was coded and analyzed using GraphPad prism 7 software and Excel. The software R was used to calculate the correlation coefficient between the results of the detection of ESBL+ on agar and by the effect of the double synergy. The results showed that sixty-three (63) bacterial strains were isolated from the 130 samples, of which the dominant species was *Chryseomonas luteola* (10/63). The kitchen samples were the most contaminated with 36.50%. More than 40% of the isolates were resistant to at least three different classes of antibiotics. Also, blaSHV gene was detected in 33.33% (21/63) of the isolates and in all isolates of *Pseudomonas aeruginosa* (5/5%). 11.11% (7/63) of isolates were virulent with dominance of the *fim*H gene, especially with *Escherichia coli* (83.33%). The kitchen samples showed a high prevalence of ESBL-producing strains with *fim*H gene. This raises the problem of non-compliance with hygiene rules in community cooking and food handling.

## 1. Introduction

Antimicrobial resistance has become a major threat to global public health in the 21^st^ century [1]. It is estimated that, by 2050, 10 million deaths per year and up to $100 trillion of global economic loss will occur if the problem of antimicrobial resistance is not immediately resolved [2]. In recent years, in the Enterobacteriaceae family in particular, there has been increasing evidence of a high prevalence of multiresistant Gram-negative bacteria. The use of antibiotics in human medicine, veterinary medicine, and agriculture has been the main selective pressure for the emergence of drug-resistant bacteria [3].

The emergence and rapid spread of extended spectrum *β*-lactamase- (ESBL-) producing bacteria poses a threat to the population, not only in developing countries, but also in developed countries, as ESBLs may degrade not only penicillin, but also other classes of *β*-lactams. In addition, most ESBL-producing bacteria are multiresistant [4]. The increased presence of Enterobacteriaceae producing ESBL in non-clinical environments such as food, animal husbandry, and agriculture and in different aquatic environments has been shown in several studies [5–7]. In addition, multiresistant enterobacteria have been isolated from healthy individuals [8], suggesting that the community may serve as a reservoir. It is known that the intestinal tract is an excellent reservoir for these bacteria and that colonized people may be infected later. In fact, the presence of *Escherichia coli* producing CTX-M has been reported in healthy children [9], which shows the potential risk of spreading *β*-lactamase-mediated resistance.

The dissemination of *β*-lactamase-encoding genes in different geographical regions may partly be due to the fact that many resistance genes are carried on self-transmissible or mobile plasmids capable of spreading horizontally between and within species [3]. During gene transfer, resistance mechanism to other classes of antibiotics can easily be combined [10]. There are different types of *β*-lactamase enzymes present in bacteria inhabiting diverse environments and exposed to different selection pressures [11]. The virulence factors of Enterobacteria have an important role in the development of infections [12]. The molecular characteristics and functions of these virulence factors have been determined [13]. The most likely theory is that strains of pathogenic enterobacteria evolved primarily from non-pathogenic strains by acquiring virulence genes (by horizontal transfer of plasmid, bacteriophage, transposon, and pathogenicity located at chromosomal loci), which provides increased adaptive capacity to new niches and allows pathogens to increase their ability to cause wide range of infections [14, 15]. Moreover, evolution of virulence and resistance occurred at different time points. Virulence has evolved over millions of years, but resistance is a new phenomenon that has occurred over the past few decades (after introduction of antibiotics). As aquatic bacterial genetic reactors, aquatic environments promote the transmission of resistance genes virulence, and virulence-related characteristics among bacterial strains [16].

In Benin, several studies have reported the increasing evolution of the prevalence of broad-spectrum *β*-lactamase-producing enterobacteria in clinical infections [17–19]. But few studies have reported broad-spectrum *β*-lactamase-producing bacteria isolated simultaneously from external environment, kitchen environment, and food products. The current study is aimed at providing scientific knowledge regarding the spread of broad-spectrum *β*-lactamase-producing enterobacteria in different environments in Benin, and also at characterizing the resistance genes and determining their virulence factors. Such data will be used to better understand clinically observed antibiotic resistance. The study will also serve as a basis for recommendations to combat antibiotic resistance.

## 2. Material and Methods

### 2.1. Zone and Period of Study

**Figure 1:**
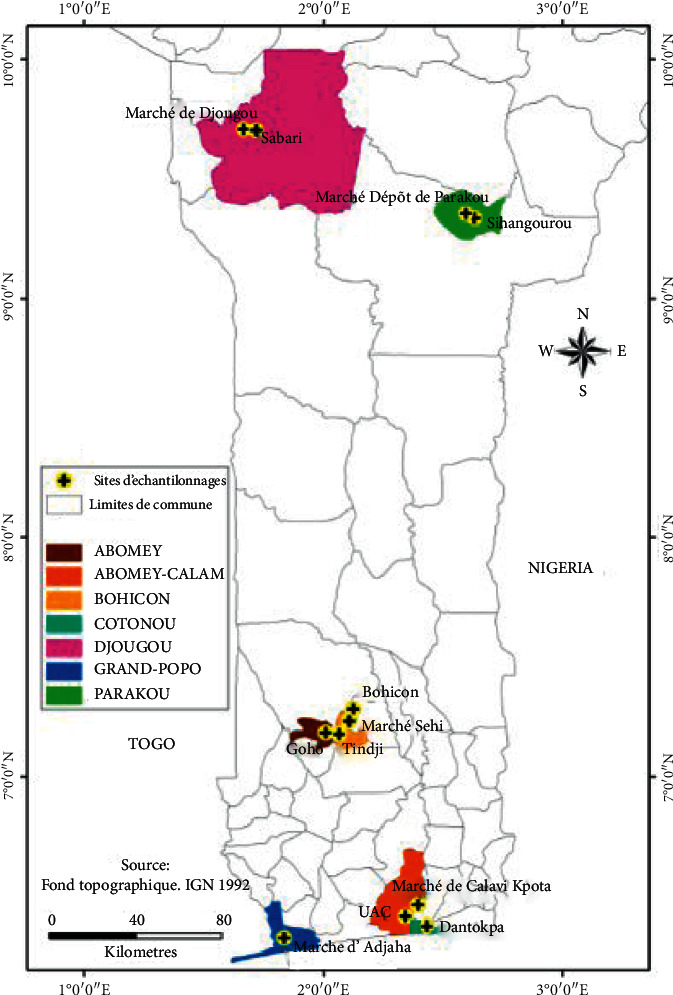
Location map of collection's areas.

The study was carried out mainly in the South of Benin, and in some towns in Northern Benin.^2^. In Southern Benin, the average annual temperature is 28°C and the humidity varies between 69 and 97% [19]. The phytogeographic districts covered by this study are Coastal, Pobè, Ouémé valley, Plateau, Zou, Borgou, and Donga. The study was done in December 2020. The samples were collected from community kitchen environment (cloth, sink, sponge, soap, and bench), external environments (sediment/mud, sewage, feces of animal's cattle, pig, and chicken), and foods products (crabs, fresh shrimps, dried fishes, fermented fishes, tomatoes, and leafy vegetable) (Figure 1).

### 2.2. Collection of Samples

Sampling was done randomly, depending on sample availability. Thirty (30) samples from the community kitchen environment were collected by swabbing. Thirty (30) sediments/mud, nine (9) animal feces, and forty-two (42) food products were collected in sterile stomach bags. Nineteen (19) sewage samples were collected in sterile one-liter bottles. A total of 130 samples were collected from the local markets of Dantokpa, Grand-Popo, Abomey-Calavi, the University of Abomey-Calavi, Bohicon, Abomey, Parakou, and Djougou (Table 1).

### 2.3. Bacteriological Analysis

#### 2.3.1. Seeding

*Sewage Samples*. After homogenization, 1 ml of each sample was taken and added to 9 ml of sterile distilled water contained in a screw tube for the preparation of 10^−1^ dilution. Successive decimal dilutions were made up to 10^−3^and 100 *μ*l of each dilution was then inoculated on McConkey and ChromID^™^ ESBL solid media (Biomerieux, France).

*Solid Samples*. Previously, all food samples were ground in a sterile mortar. 9 ml of sterile distilled water was added to 1 g of each solid sample (sediment/sludge, animal feces, and crushed food item) contained in a screw tube (stock solution). After homogenization through a vortex, 1 ml of the stock solution (10^−1^ dilution) was added again to 9 ml of distilled water (10^−2^ dilution). Successive decimal dilutions were made up to 10^−4^. The last three dilutions (10^−2^, 10^−3^, and 10^−4^) were then seeded on McConkey solid media and ChromID^™^ ESBL (Biomerieux, France).

*Community Kitchen Samples*. Each *community* kitchen sample swab was cultured on McConkey and ChromID^™^ ESBL solid media by streaking with sterile loop (Biomerieux, France).

### 2.4. Incubation

All previously prepared McConkey and ChromID^™^ ESBL agars (Biomerieux, France) were incubated at 37°C for 24 h.

### 2.5. Biochemical Identification

After 24 h incubation, Gram staining was performed on all characteristic colonies on McConkey. Each selected characteristic colony (Gram-negative bacillus) was removed and seeded on Mueller-Hinton solid medium (MH). The MH agar plates were then incubated at 37°C for 24 h. Gram controls, oxidase test, and the identification by the gallery API20E tests were made after.

### 2.6. Susceptibility of Strains to Antibiotics

An antibiogram was performed for each bacterial sample isolated by method of disk diffusion using Mueller-Hinton agar II medium in accordance with the recommendations of the Antibiogram Committee of the French Society of Microbiology [20]. The antibiotics tested against the isolates were amoxicillin (AMX) (25 *μ*g), gentamicin (GEN) (10 *μ*g), ceftriaxone (CRO) (30 *μ*g), cefotaxime (CTX) (30 *μ*g), ertapenem (ETP) (10 *μ*g), imipenem (IPM) (10 *μ*g), ciprofloxacin (CIP) (5 *μ*g), amoxicillin + clavulanic acid (AMC) (30 *μ*g), and aztreonam (ATM) (30 *μ*g). They were acquired from Sigma Aldrich.

The reference strain (*E*. *coli* ATCC 25923) and the strains to be tested were previously subcultured on the MH agar and incubated for 24 h at 37°C in order to provide young colonies. For each strain inoculum correspondent standard McFarland 0.5 was prepared. Using a sterile swab, the inoculum obtained was cultured by swabbing on dried MH II medium. The spreading was rotated three times in order to obtain a homogeneous distribution of the inoculum. The pre-impregnated antibiotic discs were then gently deposited on the surface of the agar with a disc dispenser. The agar plates were incubated for 24 h at 37°C.

A double disc diffusion method was used for the phenotypic confirmation of ESBL production according to the guidelines described by the Clinical Laboratory Standards Institute [21]. *Klebsiella pneumonia* (ATCC 700603) was used as the standard strain.

### 2.7. Molecular Detection of Virulence-Associated and ESBL Genes

The DNA of all isolates was extracted using the Qiagen red extraction kit. The isolates were tested for different virulent genes using standard PCR with six sets of specific primer pairs (Table 2). The reaction mixture consisted of 9.5 *μ*L of water without DNA, 12.5 *μ*L of the 2XPCR Master Mix reagent, 0.5 *μ*L of each primer pair, and 2 *μ*L of bacterial DNA.

The virulence genes that were targeted for amplification by PCR were *iss*, *fim*H, *Stx*-1, *Stx*-2, biofilm, and *Pap*C, whereas the ESBL genes targeted for amplification were blaTEM, blaSHV, blaCTX-M1, blaCTX-M2, blaCTX-M9, and blaCTX-M15.

The amplification of bla_TEM_, bla_SHV_, bla_CTX-M1_, bla_CTX-M2_, bla_CTX-M9_, and bla_CTX-M15_ genes was carried out using the method described in [22, 23]. The amplification of fimH and iss genes was performed using the method described in [24]. Regarding PapC gene, its amplification was also carried out using the method previously described in [25]. The amplification conditions of the other genes (Stx-1, Stx-2, and Biofilm) involved initial denaturation at 94°C for 30 sec, followed by 30 cycles at 94°C for 30 secs of denaturation at 68°C for 1 min of hybridization, at 68°C elongation for 1 min. Final extension was carried out at 68°C for 5 min and maintained at 4°C. Amplification products were separated by 2% agarose gel electrophoresis with 5 *μ*g/ml red gel and a 100 bp DNA ladder as a molecular weight marker. The migration was carried out at a scale of 90 V/cm for 20 min. Amplification bands were visualized and photographed under ultraviolet light (UV).

### 2.8. Statistical Analyses

The data collected was coded and analyzed using GraphPad prism 7 software. Phenotypic identification methods were performed independently in triplicate. The software R was used to calculate the correlation coefficient between the results of the detection of ESBL + on agar and by the effect of the double synergy.

## 3. Results

### 3.1. Culture on McConkey Agar

Of the 130 samples analyzed, we were able to isolate 63 bacterial strains, all Gram-negative bacilli. The kitchen environment samples contained 36.50% (23/63) of the isolated bacterial strains, followed by external environmental samples (33.33%) (21/63) whereas the samples from food products were 30.15% (19/63).

### 3.2. Culture on ChromID^™^ ESBL Agar

Samples were cultured on both McConkey Agar and ChromID^™^ ESBL Agar. The latter provided us with preliminary data on strains that produce *β*-lactamase. Of the 130 samples analyzed, 21 *β*-lactamase-producing strains were isolated. Furthermore, 47.61% *β*-lactamase-producing strains were isolated from community kitchen samples, 42.85% from external environmental samples, and 9.52% from food products.

### 3.3. Identification of Isolates

**Figure 2:**
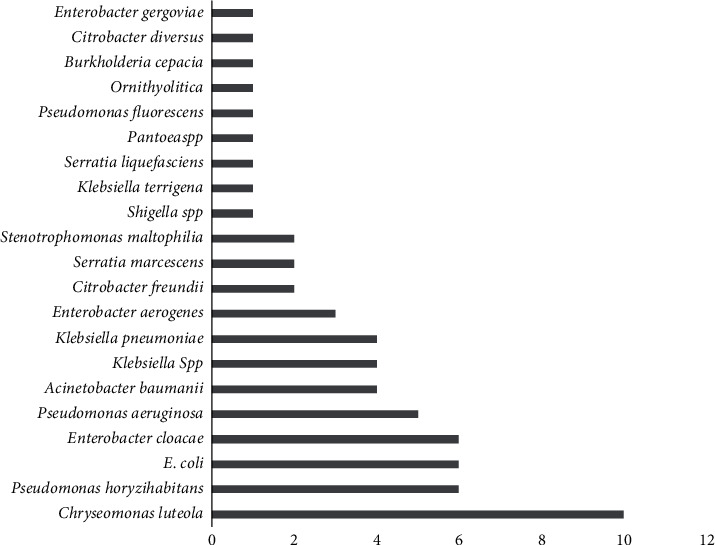
Distribution of isolated bacterial species.

After isolation, a total of 63 strains were identified and there were about 21 bacterial species. As shown in Figure 2, there is a dominance of *Chryseomonas luteola* (10/63), followed by *Pseudomonas oryzihabitans* (6/63), *Enterobacter cloacae* (6/63), and *E*. *coli* (6/63).

### 3.4. Antimicrobial Susceptibility

Table 3 shows the details of the antibiotic resistance profile of the identified strains. Of the 63 isolates, 59 (93.65%) were resistant to Amoxicillin and 54 (85.71%) to Cefotaxime, with relatively low resistance to Imipenem (16) (25.39%), Gentamicin (9) (14.28%), and Ciprofloxacin (5) (7.93%). The multidrug resistance analysis of the strains showed that there were 27.57% isolates that were resistant to at least 5 antibiotics, 19.95% isolates resistant to 4 antibiotics, and 16.81% isolates resistant to 3 antibiotics. We noted that there are two isolates, *P*. *aeruginosa* and *P*. *fluorescens*, that were resistant to all 9 antibiotics tested.

### 3.5. PCR Detection of Virulence-Associated Genes of Isolates

**Figure 3:**
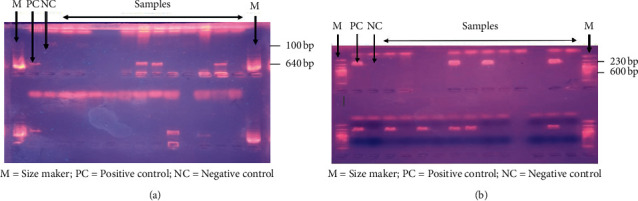
(a) Agarose gel of *fim*H gene (640 bp) after PCR. (b) Agarose gel of bla_SHV_ gene (230 bp) after PCR.

Six genes (*iss*, *fim*H, *stx*1, *stx*2, biofilm, and *Pap*C) of virulence were investigated in the genome of the 63 identified isolates. Only the *fimH* gene was detected as indicated in Table 4.  Figure 3(a) shows the agarose gel of *fim*H after PCR.

### 3.6. PCR Detection of ESBL Genes of Isolates

Of six ESBL genes (bla_TEM_, bla_SHV_, bla_CTX-M1_, bla_CTX-M2_, bla_CTX-M9_, and bla_CTX-M15_), only the Bla_SHV_ gene was found in the genome of 21 isolates. These isolates comprised *Pseudomonas aeruginosa* (100%) (5/5), followed by *Pseudomonas fluorescens (100%) (1/1) and K*. *pneumoniae* (75%) (3/4). All the strains isolated on ChromID^™^ ESBL agar and whose double synergy was observed during antibiotic susceptibility tests were also positive for the detection of the bla_SHV_ gene (*r* = 1, *P* < 0.001**)**.  Figure 3 shows the agarose gel of bla_SHV_ after PCR (Table 5).

## 4. Discussion

### 4.1. Prevalence of Pathogens in the Samples

Of the 130 samples analyzed, we were able to isolate 63 bacterial strains, all Gram-negative bacilli. The kitchen environment samples contained 36.50% (23/63) of the isolated bacterial strains, followed by external environmental samples (33.33%) (21/63), whereas the samples from food products were 30.15% (19/63). In addition, 47.61% of the bacteria isolated from cooking samples are ESBL producers. These results show that there is a high risk of contamination of food products in these kitchen environments if the appropriate practice of hygiene is not maintained. A report from a study on ESBL-producing Enterobacteriaceae isolated from food prepared in the kitchen of a hospital center [26] showed that 8.04% of foods from the hospital kitchen were contaminated with ESBL-producing strains. This low prevalence reflects the minimum respect of hygiene rules in the kitchen of the hospital. Thus, the risk of contamination was lower in this environment, but this should not be extrapolated to the community kitchen environment, where safety standards are probably less rigorously respected [27].

Of 48 external environmental samples (wastewater and sediment), 42.85%% of the strains produced ESBL. This proportion is relatively lower than that obtained by [28], where 52.69% of the ESBL-producing Enterobacteriaceae were reported. However, these results are relatively high than those obtained by [29], where 15.47% of the ESBL-producing bacteria were noted. These different results show that there is a real problem of environmental contamination in Benin. Several studies have been conducted on the isolation of ESBL-producing *E*. *coli* from rivers. The human and animal ESBL-producing *E*. *coli* in various river waters may be related to the nature of their watersheds, the intensity of antibiotic use, and the relative degree of fecal contamination by humans and animals. Clonal groups similar to those found in human infections have also been isolated from contaminated rivers adjacent to large cities [30, 31].

The prevalence of ESBL-producing bacteria was 13.75% from fecal animal samples. Also, this same proportion was obtained in piglet feces in India [32]. These results reflect the risk of contamination of slaughter meat and food products of animal origin. Of the 43 food samples, there is a prevalence of 9.52% of ESBL-producing bacterial strains. This result is relatively small compared to the 20.75% of Gram-negative *Bacillus* (GNB) ESBL producers obtained by [33] from hospital samples in Benin.

### 4.2. Antimicrobial Resistance

In this study, 63 bacterial strains from 21 species were isolated. The most identified bacterial species is *C*. *luteola* (10/63). Antibiotic susceptibility analysis showed high resistance of bacterial isolates to penicillin, cephalosporins, and aztreonam. This high resistance of the isolates coincides with the results obtained by [28] that was conducted on environmental samples and food in Spain. In addition, it was noted that the isolates showed significant resistance to *β*-lactamase inhibitors such as amoxicillin + clavulanic acid (70.39%). These results coincide with those from [34], where resistance of strains to beta-lactamase inhibitors is noted. This suggests that there is an increasing need to encourage the use of alternative beta-lactamase inhibitors. For the multidrug resistance analysis of bacterial isolates, 10 (16.81%) of the isolates were resistant to at least three different families of antibiotics. These results further emphasize the problem of multidrug-resistant bacteria in the environment which may threaten both human and animal health. The resistance of a high proportion of bacterial strains to carbapenems (Imipenem (IPM): 25.39%; Ertapenem (ETP): 61.90%) is a serious public health problem, as these antibiotics are the most effective of the beta-lactams against Gram-positive and Gram-negative bacteria, with a broad spectrum of activity [35].

### 4.3. ESBL and Virulence-Associated Genes Detection

Phenotypic detection of bacterial ESBL-producing strains was carried out by the double synergy technique. Only 21/63 of isolates were ESBL-producing; a correlation (*r* = 1) was noted between the two phenotypic detection techniques of ESBL-producing isolates. This involves the culture of samples on ChromID^™^ ESBL agar and the search for the double synergistic effect during the antibiotic susceptibility test. The presence of the bla_SHV_ gene in the genome of 33.33% of isolates confirms this correlation. *P*. *aeruginosa* (100%) was the most predominant among these strains with ESBL genes. However, among the ESBL genes sought, only the bla_SHV_ gene was found in the ESBL-positive isolates. The bla_SHV_ carrying organisms have also been detected in a variety of environmental and food samples [36]. Despite the high cefotaxime resistance observed, the bacterial strains were negative to bla_CTX-M_, suggesting that other genes may be involved in the resistance mechanism.

The search for virulence genes also showed the presence of a single virulence gene in the isolates; the *fim*H gene, also known as the fimbriae adhesion gene type 1 (*fim*H), recorded the prevalence of 11.11% in this study. *E*. *coli* (83.33%) was the most predominant bacterial among this group. It is believed that *fim*H contributes to the protection of *E*. *coli* from heterophilous hosts [37]. Its role in the virulence of Avian Pathogenic *E*. *coli* strains remains controversial and is the subject of conflicting reports [38]. Other studies found higher occurrences of the *fim*H gene, particularly those from [39], which found a prevalence of 98.1% among 524 Avian Pathogenic *E*. *coli* isolates.^*∗*^

## 5. Conclusion

This study allowed us to characterize the multidrug resistance, virulence, and ESBL production of bacterial strains isolated from the external environment, food products, and community kitchen samples. Only 33.33% of the isolates were ESBL-positive and 11.11% harboured the virulence *fim*H gene. Only the bla_SHV_ gene was present in the genome of isolated strains. The community kitchen samples showed a high prevalence of ESBL-producing bacteria and *fim*H gene. This raises the problem of non-compliance with hygiene rules in community kitchen.

## 6. Limitations

This study presents some limitations. We did not identify exact bla_SHV_ alleles due to PCR limitation. Secondly, we could not confirm carbapenem resistance genotypically. In addition, we could not detect other *β*-lactamase genes and virulence factors that may be present. Future studies including whole genome sequencing could help to address these limitations.

## Figures and Tables

**Table 1:** Distribution of samples according to their nature and origin.

Sample origin	Type of sample	Location	Positive samples
Community kitchens	Cloth	Abomey-Calavi	**6**
	Sink	Abomey-Calavi	**12**
	Bench	Abomey-Calavi	**3**
	Sponge	Abomey-Calavi	**6**
	Soap	Abomey-Calavi	**3**

Sediments/muds	Sediment of waste water (from Kpahou's farm)	Ouidah	**3**
	Gutter sediment	Abomey-Calavi street	**6**
	Lake sediment	Abomey-Calavi market	**6**
	Mud	Grand-Popo market	**3**
	Sediment of little beach	Grand-Popo	**6**
	Sinangourou bridge waste water's sediment	Sinangourou, Parakou	**1**
	Sewage sediment from the Sabari drainage canal	Sabari, Djougou	**2**
	Drainage canal crossing Abomey	Goho, Abomey	**1**
	Drainage canal crossing Bohicon	Bohicon	**1**
	Drainage canal crossing Tindji	Tindji	**1**

Sewage	Water from River	Grand-Popo	**3**
	Sewage from market	Abomey-Calavi	**3**
	Sewage from market	Dantokpa (Cotonou)	**3**
	Sewage from market	Grand-Popo	**3**
	Sewage from University of Abomey-Calavi	Abomey-Calavi	**3**
	Sinangourou bridge's waste water	Parakou, Sinangourou	**1**
	Waste water drainage system crossing Djougou	Sabari, Djougou	**2**

Feces of animals	Feces of cattle from market	Grand-Popo	**3**
	Feces of pig from market	Grand-Popo	**3**
	Feces of chicken from market	Grand-Popo	**3**

Foods	Crabs from market	Grand-Popo	**3**
	Leafy vegetable from market	Grand-Popo	**3**
	Fresh shrimp from market	Grand-Popo	**3**
	Dried fishes from market	Grand-Popo	**3**
	Fresh tomatoes from market	Grand-Popo	**3**
	Fresh shrimp from market	Dantokpa (Cotonou)	**3**
	Dried fishes from market	Dantokpa (Cotonou)	**3**
	Fermented fishes from market	Dantokpa (Cotonou)	**3**
	Fresh tomatoes from market	Dantokpa (Cotonou)	**3**
	Fresh tomatoes from market	Djougou	**4**
	Dried fishes from market	Abomey	**3**
	Leafy vegetable from market	Parakou	**4**
	Soja cheese	Bohicon	**5**

Total			**130**

**Table 2:** Primers of virulence genes and desired ESBL genes in the genome of isolates.

Genes	Primer	Sequence (5'-3')	Amplicon size (bp)	Reference (s)
Virulence				
*Pap*C	F	GACGGCTGTACTGCAGGGTGGCG	328	[3]
	R	ATATCCTTTCTGCAGGGATGCAATA		
*fim*H	F	TACTGCTGATGGGCTGGTC	640	
	R	GCCGGAGAGGTAATACCCC		
*iss*	F	GGCAATGCTTATTACAGGATGTGC	260	
	R	GAGCAATATACCCGGGCTTCC		
Biofilm	F	GATTCAATTTTGGCGATTCCTGC	225	
	R	TAATGAAGTCATTCAGACTCATCC		
Stx1	F	CGCTGAATGTCATTCGCTCTGC	302	
	R	CGTGGTATAGCTACTGTCACC		
Stx2	F	CCTCGGTATCCTATTCCCGG	516	
	R	CTGCTGTGACAGTGACAAAACGC		

ESBL				
bla_CTX-M1_	F	GGTTAAAAAATCACTGCGTC	863	[4]
	R	TTGGTGACGATTTTAGCCGC		
bla_CTX-M2_	F	ATGATGACTCAGAGCATTCG	865	
	R	TGGGTTACGATTTTCGCCGC		
bla_CTX-M9_	F	ATGGTGACAAAGAGAGTGCA	869	
	R	CCCTTCGGCGATGATTCTC		
bla_CTX-M15_	F	CACACGTGGAATTTAGGGACT	995	
	R	GCCGTCTAAGGCGATAAACA		
bla_TEM_	F	ATGAGTATTCAACATTTCCGC	856	
	R	CAATGCTTAATCAGTGAGG		
bla_SHV_	F	AAGATCCACTATCGCCAGCAG	230	
	R	ATTCAGTTCCGTTTCCCAGCGG		

**Table 3:** Antimicrobial susceptibility profiles of isolates.

Antibiotics	% of resistance
Ceftriaxone (CRO)	52.38
Cefotaxime (CTX)	85.71
Amoxicillin (AMX)	93.65
Gentamicin (GEN)	14.28
Ciprofloxacin (CIP)	7.93
Imipenem (IPM)	25.39
Ertapenem (ETP)	61.90
Amoxicillin + clavulanic acid (AMC)	57.14
Aztreonam (ATM)	44.44

**Table 4:** Distribution of virulence-associated genes in different isolates.

Isolates	No.	No. (%) of virulence-associated genes					
		Iss	fimH	Biofilm	PapC	stx1	stx2
*Acinetobacter baumanii*	4	0	0	0	0	0	0
*Pseudomonas horyzihabitans*	6	0	0	0	0	0	0
*Pseudomonas aeruginosa*	5	0	1 (20)	0	0	0	0
*Pseudomonas fluorescens*	1	0	0	0	0	0	0
*Klebsiella pneumonia*	4	0	0	0	0	0	0
*Klebsiella terrigena*	1	0	0	0	0	0	0
*Klebsiella ornithynolitica*	1	0	0	0	0	0	0
*Serratia marcescens*	2	0	0	0	0	0	0
*Serratia liquefasciens*	1	0	0	0	0	0	0
*Chryseomoas luteola*	10	0	0	0	0	0	0
*Enterobacter aerogenes*	3	0	1 (33.33)	0	0	0	0
*Enterobacter cloacae*	6	0	0	0	0	0	0
*Stenotrophomonas maltophilia*	2	0	0	0	0	0	0
*Escherichia coli*	6	0	5 (83.33)	0	0	0	0
*Shigella spp*	1	0	0	0	0	0	0
*Pantoea spp*	1	0	0	0	0	0	0
*Burkholderia cepacian*	1	0	0	0	0	0	0
*Klebsiella Spp*	4	0	0	0	0	0	0
*Citrobacter freundii*	2	0	0	0	0	0	0
*Citrobacter diversus*	1	0	0	0	0	0	0
*Enterobacter gergoviae*	1	0	0	0	0	0	0
Total	63	0	7 (11.11)	0	0	0	0

**Table 5:** Distribution of ESBL genes in different isolates.

Isolates	ESBL genes (%)						
	No	Bla_TEM_	Bla_SHV_	Bla_CTX-M1_	Bla_CTX-M2_	Bla_CTX-M9_	Bla_CTX-M15_
*Acinetobacter baumanii*	4	0	0	0	0	0	0
*Pseudomonas horyzihabitans*	6	0	0	0	0	0	0
*Pseudomonas aeruginosa*	5	0	5 (100)	0	0	0	0
*Pseudomonas fluorescens*	1	0	1 (100)	0	0	0	0
*Klebsiella pneumonia*	4	0	3 (75)	0	0	0	0
*Klebsiella terrigena*	1	0	0	0	0	0	0
*Klebsiella ornithynolitica*	1	0	0	0	0	0	0
*Serratia marcescens*	2	0	1 (50)	0	0	0	0
*Serratia liquefasciens*	1	0	0	0	0	0	0
*Chryseomoas luteola*	10	0	0	0	0	0	0
*Enterobacter aerogenes*	3	0	3 (100)	0	0	0	0
*Enterobacter cloacae*	6	0	3 (50)	0	0	0	0
*Stenotrophomonas maltophilia*	2	0	1 (50)	0	0	0	0
*Escherichia coli*	6	0	2 (33.33)	0	0	0	0
*Shigella spp*	1	0	1 (100)	0	0	0	0
*Pantoea spp*	1	0	1 (100)	0	0	0	0
*Burkholderia cepacian*	1	0	0	0	0	0	0
*Klebsiella Spp*	4	0	0	0	0	0	0
*Citrobacter freundii*	2	0	0	0	0	0	0
*Citrobacter diversus*	1	0	0	0	0	0	0
*Enterobacter gergoviae*	1	0	0	0	0	0	0
Total	63	0	21 (33.33)	0	0	0	0

## Data Availability

All data generated or analyzed during this study are included in this published article.
